# *Staphylococcus aureus* and Influenza A Virus: Partners in Coinfection

**DOI:** 10.1128/mBio.02068-16

**Published:** 2016-12-13

**Authors:** Michelle E. Mulcahy, Rachel M. McLoughlin

**Affiliations:** Host-Pathogen Interactions Group, School of Biochemistry and Immunology, Trinity Biomedical Sciences Institute, Trinity College Dublin, Dublin, Ireland

## Abstract

Nasal carriage of *Staphylococcus aureus* is a significant risk factor for secondary staphylococcal pneumonia in influenza A virus (IAV)-infected hosts. However, little research has been undertaken to define the environmental and physiological changes that cause *S. aureus* to shift from commensal to pathogenic organism in this setting. The ability of virus-driven danger signals to cause *S. aureus* to transition from commensalism to pulmonary infection was explored in a recent study by Reddinger et al. R. M. Reddinger, N. R. Luke-Marshall, A. P. Hakansson, and A. A. Campagnari, mBio 7(6):e01235-16, 2016, http://dx.doi.org/10.1128/mBio.01235-16. The authors report that physiological host changes, including febrile temperature and a combination of host stress response signals, caused *S. aureus* biofilms to disperse from the nasal environment and cause active pulmonary infection. This commentary discusses the new finding in light of the current understanding of the mechanisms behind staphylococcal coinfection with IAV. In addition, it considers the mechanisms behind staphylococcal dispersal in this model. Overall, the study indicates that interkingdom signaling may occur following IAV infection and this likely contributes to sensitizing the IAV-infected host to secondary staphylococcal pneumonia.

## COMMENTARY

*Staphylococcus aureus* is a frequent perpetrator of secondary bacterial pneumonia following influenza A virus (IAV) infection. In recent years, methicillin-resistant *S. aureus* (MRSA) strains, such as USA300, have been implicated in severe or fatal cases of secondary pneumonia in otherwise healthy individuals who have contracted IAV (1, 2). *S. aureus* is also a common resident of the human microbiome and is present persistently and asymptomatically in the anterior nares of 20% of the healthy human population, while the remainder have the potential to be intermittently colonized (3). Persistent nasal carriers of *S. aureus* are predisposed to invasive disease, including secondary staphylococcal respiratory infection (4–7); *S. aureus* may be aspirated from the nose into the lung, with the potential to cause respiratory infection in a host made susceptible by the presence of IAV.

The majority of research on secondary staphylococcal respiratory infection has focused on IAV-elicited host immune factors that increase host susceptibility to secondary bacterial pneumonia due to an impaired or insufficient immune response to fight bacterial infection. This phenotype is primarily attributed to the production of interferons (IFNs), which trigger the induction of IFN-stimulated genes (ISGs) and the production of antiviral proteins, which are necessary to inhibit viral replication (8). Concurrent with their antiviral effect, however, IFN production can inhibit a number of important antibacterial immune responses. For example, type I IFNs selectively inhibit the production of the important neutrophil-recruiting chemokines KC/CXCL1 and Mip2/CXCL2 in mouse lungs during secondary *S. pneumoniae* infection, leading to attenuated neutrophil responses (9).

The IAV-mediated host immune response may also influence nasal carriage of *S. aureus* in an infected individual. Type I IFN inhibits the interleukin-23 (IL-23)-dependent induction of Th17 immunity in the lung (10). This results in lower levels of IL-17-producing CD4^+^ and γδ T cells in the lung and, consequently, less IL-17 and IL-22 production, preventing efficient clearance of bacteria. IL-17 and IL-22 are both important determinants of *S. aureus* nasal carriage *in vivo*; IL-17 is important for neutrophil-mediated clearance of *S. aureus* from the nose (11), while IL-22 controls local antimicrobial peptide production and staphylococcal ligand expression (12). Consequently, it is likely that IAV-mediated Th17 suppression affects *S. aureus* nasal carriage, as well as secondary infection.

Consistent with this, a recent study has demonstrated the effect of IAV infection on the composition of the nasal microbiome. Interestingly, these effects were attributable to IAV-driven activation of type III IFN signaling, as opposed to type I IFN responses (13). IAV-infected mice harbored significantly more upper-respiratory commensal bacteria than healthy mice, in combination with an increase in the relative abundance of murine commensal staphylococci. This correlated with higher type III IFN expression in the upper airway of IAV-infected mice. *S. aureus*-colonized mice that were then infected with IAV displayed increased bacterial burdens in both the nose and the lungs compared with those in mice treated with PBS. This change in microbial abundance in the upper respiratory cavity and subsequent onset of secondary staphylococcal superinfection has been linked to the induction of virus-triggered type III IFN signaling, which results in altered IL-22 responses that lead to impaired expression of antimicrobial peptides like Reg3γ and lipocalin in the nasal cavity.

The bacterial factors involved in the transition of *S. aureus* from commensal to pathogenic organism in response to environmental stimuli during IAV infection are largely unknown. A recent study by Reddinger et al. (14) explores the transition of *S. aureus* from normal commensal to causative agent of secondary pneumonia in response to physiological changes in the host brought about by IAV infection. Reddinger et al. observed that physiological changes associated with viral infection, including febrile temperature, release of nutrients, and exogenous ATP, caused *S. aureus* biofilms to disperse from human bronchial epithelial cells *in vitro* and from the nasal cavity to the lung *in vivo*. *S. aureus*-colonized mice that were subsequently infected with IAV retained significantly more *S. aureus* bacteria in the nose and lungs and also developed secondary staphylococcal pneumonia more frequently than colonized control mice, indicating that IAV infection causes this shift from asymptomatic colonization to invasive disease by dispersing *S. aureus* biofilms within the nasal cavity. This study concludes that physiological changes in the host elicited by viral infection drive *S. aureus* to transition from an asymptomatic commensal organism to an infectious agent that can cause invasive disease.

In contrast to studies that have examined changes in the host immune response brought on by IAV infection and subsequent failure to prevent *S. aureus* dissemination to the lungs, this study focuses on the relatively unknown direct changes in the bacterial response to IAV infection. Furthermore, while the majority of other coinfection models allow IAV infection to take hold and manipulate the host response before introducing a secondary infection, this paper mimics the natural physiological timeline of secondary staphylococcal pneumonia, as mice are colonized asymptomatically before viral infection takes hold.

The results obtained in the study by Reddinger et al. support the concept that subsequent IAV infection leads to a shift in bacterial burden to the respiratory tract via biofilm dispersal from the nose (14). However, it is unclear whether this effect is specifically due to IAV-induced danger signals. Displacement of loosely adherent bacteria from the nose to the lungs by the physical administration of IAV following bacterial inoculation cannot be ruled out without including a bacterium-inoculated, PBS-treated control. Additionally, examining the effects of danger signals induced by alternative viral infections would determine the specificity of the observed interactions. For example, do danger signals elicited by other respiratory viruses—particularly a signal as common as febrile temperature—also lead to effective biofilm dispersal to the lungs, or is this effect unique to IAV? Furthermore, possible virus-induced epithelial cell damage that occurs in the nasopharynx following IAV infection may lead to higher levels of bacterial adherence in the trachea, aiding in bacterial dissemination. In a model of secondary pneumococcal infection, the respiratory tracts of IAV-infected mice exhibited higher levels of bacterial adherence due to virus-induced desquamation of cilial and secretory tracheal cells and exposure of the basement membrane (15). IAV infection also causes damage to epithelial cell tight junctions *in vitro* (16). Epithelial damage may expose important staphylococcal attachment sites, thus facilitating increased adherence in the nasal cavity and dissemination to the lungs.

While this study shows that *S. aureus* responds to virus-induced physiological changes in a manner that initiates the dissemination process, the exact mechanisms that trigger this response are unknown. Previous studies on bacterial biofilms have demonstrated that external environmental signals, such as pH and osmolarity, as well as nutrient availability, can initiate biofilm dispersal (17). *S. aureus* biofilm dispersal in response to signals like glucose depletion involves the activation of the *agr* quorum-sensing system and is protease dependent (18). Both glucose and exogenous ATP promote staphylococcal biofilm formation, rather than dispersal, on inert surfaces *in vitro* (18, 19), but the additional factor of a live attachment surface both *in vitro* and *in vivo* likely provides alternative conditions for biofilm formation in the study by Reddinger et al. (14). Further investigation into the transcriptional changes that occur using the unique combination of stimuli coupled with the coculture of *S. aureus* with human epithelial cells employed by Reddinger et al. would be very informative.

**FIG 1:**
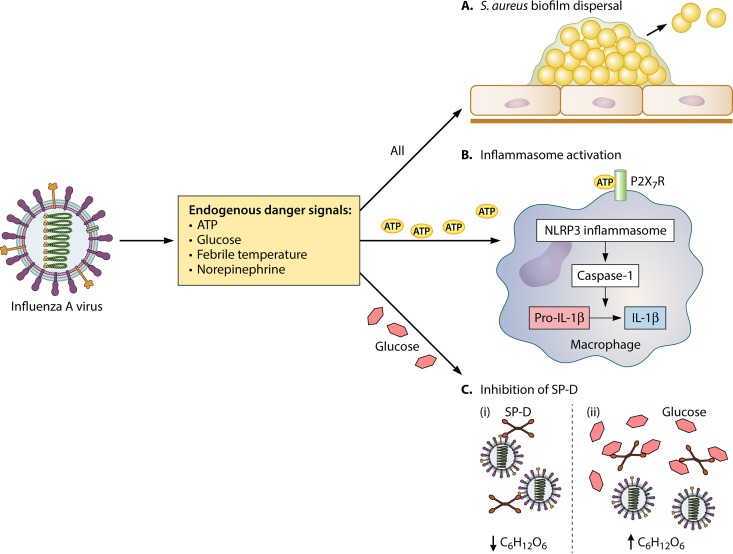
Roles of endogenous danger signals during influenza A viral infection. Endogenous danger signals, including ATP, glucose, norepinephrine, and febrile temperature ranges, are elicited from damaged cells following influenza A virus (IAV) infection. These danger signals influence the infective potential of commensal *Staphylococcus aureus*, as well as potentially manipulating innate immune responses. (A) A combination of danger signals initiates dispersal of *S. aureus* biofilms in the nasal cavity, leading to dissemination of the bacteria from the nasal epithelium to the lungs. (B) ATP can trigger the innate immune response against IAV by activating the NLRP3 inflammasome in macrophages, leading to secretion of IL-1β and the initiation of a proinflammatory response. (C) High levels of glucose can negatively affect collectin-mediated immune defenses in the lung against IAV. (i) Collectin surfactant protein D (SP-D) can neutralize the infectivity of IAV by binding to oligosaccharides on viral glycoproteins. (ii) Glucose is a ligand for SP-D. Binding of glucose to SP-D acts to inhibit SP-D-mediated antiviral activity.

Reddinger et al. (14) report that danger signals elicited in the host in response to viral infection directly cause *S. aureus* to disperse and disseminate *in vivo*; however, endogenous danger signals can also directly influence the host immune response (Fig. 1). ATP release has been postulated to play a role in inflammasome activation and initiation of the innate immune response during viral infection (20), and although activation of the Nlrp3 inflammasome can be beneficial during staphylococcal surgical site infections (21), Nlrp3 induction can contribute to the severity of staphylococcal pneumonia (22). Glucose levels can also affect the innate immune response to both IAV and staphylococcal pneumonia; higher glucose levels in diabetic mice infected with IAV led to more severe outcomes due to glucose-mediated neutralization of the antimicrobial collectin lung surfactant protein D (SP-D) (23). SP-D is vital for an effective innate immune response during both *Haemophilus influenzae* and streptococcal lung infection (24), and mice deficient in both SP-D and SP-A exhibited more severe staphylococcal pneumonia (25). These alternate roles for danger signals in directly activating innate immune pathways may also indirectly facilitate the transition of *S. aureus* to the lungs.

The question of whether *S. aureus* forms biofilms during nasal colonization has been previously debated, with evidence both supporting and contesting this notion (26). The current study provides compelling evidence of biofilm formation in the murine nasal cavity *in vivo* using scanning electron microscopy (SEM), though the specific sites of biofilm formation within the murine nasal cavity were not identified. Previous studies have identified two distinct sites for *S. aureus* colonization within the nasal cavity: the first is the anterior nares of mice, through an interaction between the *S. aureus* surface protein clumping factor B and the host ligand loricrin (27); the second is epithelial cells within the inner nasal cavity, through an interaction with the scavenger receptor SREC-I (28). Visualization of biofilm formation at distinct sites within the nasal cavity could confirm the importance of these staphylococcus-host ligand interactions in facilitating colonization if biofilm formation overlaps with sites within the nose that are rich in loricrin and/or SREC-I expression. Furthermore, visualization of biofilm changes following influenza infection could highlight the changing environment of the epithelium in response to the virus, as well as the transition of *S. aureus* from this site.

The mechanism behind the shift in balance from *S. aureus* commensalism to pathogenesis during viral infection is poorly understood. The research presented by Reddinger et al. (14) significantly advances our understanding of this process by identifying a mechanism whereby *S. aureus* actively responds to physiological changes within the host, causing dynamic dissemination from its commensal niche. This suggests that the process of secondary infection is more complex than the organism simply taking advantage of a more susceptible host and alludes to interkingdom crosstalk between IAV and the commensal microbiome of the upper respiratory tract. In the coinfection model presented in this study, it is likely that the combination of environmental changes and immune responses initiated by virus-activated host danger signals may act in tandem to create a more suitable environment for *S. aureus* secondary infection. It is clear that further investigation into the consequences of IAV infection for commensal *S. aureus* is required to uncover possible novel mechanisms controlling the onset of staphylococcal virulence.
